# Helios Expression Is Downregulated on CD8^+^ Treg in Two Mouse Models of Lupus During Disease Progression

**DOI:** 10.3389/fimmu.2022.922958

**Published:** 2022-06-16

**Authors:** Andrés París-Muñoz, Gonzalo Aizpurua, Domingo F. Barber

**Affiliations:** ^1^ Department of Immunology and Oncology, Centro Nacional de Biotecnología (CNB-CSIC), Madrid, Spain; ^2^ NanoBiomedicine Initiative, Centro Nacional de Biotecnología (CNB-CSIC), Madrid, Spain

**Keywords:** Helios, CD8^+^ Treg, lupus, autoimmunity, dendritic cells, tolerogenic DC, tolerance

## Abstract

T-cell–mediated autoimmunity reflects an imbalance in this compartment that is not restored by tolerogenic immune cells, e.g., regulatory T cells or tolerogenic dendritic cells (tolDCs). Although studies into T-cell equilibrium have mainly focused on regulatory CD4^+^FoxP3^+^ T cells (CD4^+^ Tregs), recent findings on the lesser known CD8^+^ Tregs (CD44^+^CD122^+^Ly49^+^) have highlighted their non-redundant role in regulating lupus-like disease and their regulatory phenotype facilitated by the transcription factor Helios in mice and humans. However, there are still remaining questions about Helios regulation and dynamics in different autoimmune contexts. Here, we show the absence of CD8^+^ Tregs in two lupus-prone murine models: MRL/MPJ and MRL/lpr, in comparison with a non-prone mouse strain like C57BL/6. We observed that all MRL animals showed a dramatically reduced population of CD8^+^ Tregs and a greater Helios downregulation on diseased mice. Helios induction was detected preferentially on CD8^+^ T cells from OT-I mice co-cultured with tolDCs from C57BL/6 but not in MRL animals. Furthermore, the Helios profile was also altered in other relevant T-cell populations implicated in lupus, such as CD4^+^ Tregs, conventional CD4^+^, and double-negative T cells. Together, these findings could make Helios a versatile maker across the T-cell repertoire that is capable of differentiating lupus disease states.

## Introduction

The immune system from highly developed animals is a complex and coordinated network of organs, cells, and proteins in equilibrium; the main objective of which is to maintain the host’s defenses against foreign pathogens while preventing the activation of self-reactive immune cells. Thus, optimal immune homeostasis requires implementing mechanisms that ensure specific tolerance against self-antigens (central and peripheral tolerance), and when this fails, more than 80 different autoimmune pathologies have been seen to emerge (1), including systemic lupus erythematosus (SLE) (2). Among the different adaptive immune cell populations (mainly B and T cells), autoreactive and immunosuppressive T lymphocytes have been studied in SLE, on either side of the immune balance, both as key mediators and therapeutic targets (3–5). Accordingly, in both human and murine SLE, there is an accumulation of autoreactive CD4^+^ and CD8^+^ memory T cells that cannot be counteracted by their regulatory T-cell (Treg) counterparts. Although most studies into autoimmunity and Treg function have focused largely on CD4^+^ Tregs [CD3^+^CD4^+^CD25^+^FoxP3^+^ (6, 7)], CD8^+^ Tregs are becoming increasingly relevant in autoimmunity (8, 9) and particularly in SLE (10, 11). In this regard, an emerging subpopulation of CD8^+^ Tregs (CD44^+^CD122^+^Ly49^+^) has recently been described as a non-redundant regulator of germinal center reaction and high-affinity antibody generation in mice mimicking SLE (12–14).

In contrast to CD4^+^ Tregs, the murine CD8^+^ Treg subpopulation does not express FoxP3 nor low levels of the IL-7 receptor-α (CD127), yet the homeostasis of these cells is controlled by IL-15 (15–17) and their Ly49 expression is induced post-thymically (18). However, both thymic-derived CD4^+^ Tregs and CD8^+^ Tregs do express the Helios transcription factor [encoded by *Ikzf2* gene (13)]. Indeed, how Helios expression influences the stability of the immunosuppressive phenotype of CD8^+^ Tregs has been studied (16) and, more recently, how Helios deficiency in this particular cell subset may be associated with lupus-like disease in mice and SLE in humans (12). There is evidence that Helios induction depends on by Transforming Growth Factor β (TGF-β) signaling, specifically in CD8^+^ T cells (12), but, nevertheless, there are still questions that need to be addressed to better understand how Helios is induced or repressed in the context of autoimmunity (19). Likewise, to search for new, useful molecular markers that may be suitable to be used in clinic practice, it will be necessary to explore Helios modulation not only in CD8^+^ Tregs but also in other well-known components of the immune system involved in the pathology of lupus, e.g., CD4^+^ Tregs, conventional CD4^+^ T cells (CD4^+^ Tconvs), double-negative T cells (DN T cells), and gamma delta (γδ) T cells.

In the periphery, different functional types of dendritic cells [DCs (20, 21)] play an important role in coordinating the adaptive immune response (22). As pivotal regulators of immune homeostasis, plasmacytoid (pDCs) and conventional DCs (cDCs) have already been implicated in lupus (23–25), and CD8^+^ T-cell cross-tolerance through cDCs has been proposed as a fundamental mechanism in peripheral tissues to ensure antigenic tolerance (26–28). Accordingly, DC ablation in a mouse model produces lethal autoimmunity associated with a lupus-like symptomatology (29). Nevertheless, the possible link between Helios induction of CD8^+^ T cells and the tolerogenic activity and phenotype of cDCs in autoimmunity has yet to be defined.

Here, we show the differential expression of Helios in several T-cell populations associated with the pathological changes in two different mouse models of SLE: MRL/MPJ (Murphy Roths Large: MPJ mice) and MRL/lpr (LPR mice) (30, 31). Although Helios was elevated in effector CD4^+^ Tregs and effector CD4^+^ Tconvs, correlated with disease progression, lower levels of this transcription factor were detected in CD8^+^ Tregs (virtually absent in MRL cells), DN T cells, and a population of TCRγδ^+^ B220^+^ T cells. This is especially significant when we compare these mice to healthy C57BL/6 mice (without a lupus-prone genetic background, such as that in MRL). In addition, evaluating in more detail the possible cellular contexts that influence Helios expression on CD8^+^ T cells, we observed *in vitro* that those cells from OT-I mice expressed higher levels of Helios when they were co-cultured with different types of bone marrow–derived DCs from C57BL/6 mice. Interestingly, the greatest Helios upregulation was detected on CD8^+^ T cells in presence of tolerogenic DCs (tolDCs) relative to mature (mDCs) and immature (imDCs). However, this dendritic stimulation of Helios on CD8^+^ T compartment was impaired in co-cultures from MRL animals, specially from LPR mice. In line with this point, furthermore, we identified some phenotypic alterations *in vivo* and *in vitro* in DC subsets between MPJ and LPR mice. These findings suggest that cellular environment provided by DCs could display a role controlling Helios induction on CD8^+^ Tregs and thus modulating their immunosuppressive functions. This would be consistent with data indicating that a specific CD8^+^ Treg deficiency correlates with altered DC populations.

In addition, observations from mice on a MRL/MPJ genetic background enable us to reconsider this particular spontaneous model of chronic SLE as an interesting natural lupus-prone model in which CD8^+^ Tregs are practically abolished.

## Materials and Methods

### Mice

Female MRL/MPJ, MRL/lpr, C57BL/6, and OT-I mice were maintained in pathogen-free conditions at the Centro Nacional de Biotecnología (CNB-CSIC) Animal Facility (Madrid, Spain). In the case of the MRL strains, the mice were examined twice weekly, and, when severe SLE symptoms appeared, diseased mice were sacrificed and analyzed to avoid unnecessary suffering. All animal studies were reviewed and approved by the CNB Ethics Committee for Animal Experimentation, CSIC Ethics Committee, and by the Division of Animal Protection of the regional government of the Comunidad de Madrid, in compliance with the national (RD 53/2013) and European Union legislation (directive 2010/637EU).

### Enzyme-Linked ImmunoSorbent Assay (ELISA)

Immediately after sacrifice, blood samples were extracted from the mice by cardiac puncture, and the serum was separated by centrifugation and stored at −20°C for future use. The anti-dsDNA Ig(G+A+M) antibodies were measured using a commercial ELISA kit (Cat. No. 5110, Alpha Diagnostic International) according to the manufacturer’s recommendations.

### Renal Function

The presence of protein in the urine was estimated using a urine dipstick (Combur Test^10^: Roche). After sacrifice, the kidneys of MRL/MPJ and MRL/lpr animals were collected and washed in Phosphate Buffered Saline (PBS). The fresh organs were then dehydrated in sucrose solutions (15% and then 20%), placed in a cryoprotective medium (OCT: Tissue-Tek), and maintained at −80°C. Subsequently, 4- to 10-µm kidney cryostat sections were fixed with cold acetone and blocked in PBS with 10% goat serum and 2% Bovine Serum Albumin (BSA). For IgG immunofluorescence, the sections were probed with Alexa Fluor 488–conjugated goat anti-mouse IgG (Cat. No. A-11029, Invitrogen), and, for macrophage immunofluorescence, the primary antibody used was a rat anti-mouse F4/80 (BM8, eBioscience), and it was recognized with an Alexa Fluor 488–conjugated goat anti-rat IgG secondary antibody (Cat. No. A-11006, Invitrogen). Images were taken using a Leica DMi8 S wide-field epifluorescence microscope and analyzed with ImageJ.

### Antibodies and Flow Cytometry

After sacrifice, the mouse spleens were dissected out in RPMI 1640 media and processed mechanically in a 40-µm cell strainer. After erythrocyte lysis (in an ammonium chloride solution), a single-cell suspension was obtained by passing the sample through a new 40-µm cell strainer. The cells were then counted and separated for flow cytometry experiments. In the case of flow cytometry for DCs, the cells were harvested in PBS Ethylenediaminetetraacetic acid (EDTA) (5 mM) on day 13, and the following conjugated antibodies were used: B220-efluor450 (RA3-6B2), CCR7-biotin (4B12), CD4-APCef780 (RM4.5), CD8_α_-APCef780 (53-6.7), CD11b-Alexaf700 (M1/70), CD11c-APC (N418), CD62L-APC (MEL-14), CD69-PeCy7 (H1.2F3), CD73-PeCy7 (eBioTY/11.8), CD80-PeCy5 (16-10A1), CD122-FITC (fluorescein isothiocyanate) (TM-b1), CD127-PeCy7 (A7R34), FoxP3-PE (FJK-16s), Helios-ef450 (22F6), inducible co-stimulator (ICOS)-FITC (7E.17G9), Ly49-PE (14B11), PDCA-1-PeCy7 (eBio927), RORγ(t)-PE (AFKJS-9), and TCRVα2-PE (B20.1, all from eBioscience); B220-PeCy7 (RA3-6B2), CD3-APC (145-2C11), CD3-PercP (145-2C11), CD4-APC (RM4.5), CD8_α_-APC (53-6.7), CD8_α_-PercP (53-6.7), CD44-PercP (IM7), CD49b-PE (DX5), CD86-PE (GL-1), CD335(NKp46)-APC (29A1.4), Major Histocompatibility Complex Class II (MHCII)-PE (M5/114.15.2), and PDL-1 (10F.9G2, all from BioLegend); CD11c-FITC (HL3), CD25-biotin (7D4), CD62L-PE (MEL-14), CD244.2-biotin (2B4), TCRβ-PE (H57-597), and TCRγδ-biotin (GL3, all from BD Biosciences); CD3-FITC (145-2C11), CD19-SPRD (6D5), and CD44-FITC (KM201, all from SouthernBiotech); CD244.1-biotin (REA524, Miltenyi Biotec). Secondary biotin staining was achieved with Streptavidin-APC (Beckman Coulter), Streptavidin-APCCy7 (BioLegend), or streptavidin-PercPef710 (eBioscience). Dead cells were distinguished using the Live/Dead red dye (Invitrogen) for fixed cells or Propidium Iodide (Beckman Coulter) for non-fixed cells. The gating strategies are detailed in the supplementary material. The cells were stained for FoxP3, Helios, and RORγ(t) following the instructions for the FoxP3/Transcription Factor Staining Buffer Set (eBioscience). All staining experiments were performed after incubation with an anti-CD16/32 antibody (93, SouthernBiotech) to block Fc receptors. Finally, the cells were analyzed in a Gallios flow cytometer (Beckman Coulter), and the data were analyzed with FlowJo software (TreeStar).

### Bone Marrow–Derived DC Cultures

On the basis of low-density cultures on day 0 (32), bone marrow precursors were isolated from the femurs and tibiae of healthy 8- to 12-week-old mice (C57BL/6, MRL/MPJ, and MRL/lpr) in PBS containing EDTA (5 mM) and 3% Fetal Bovine Serum (FBS). After erythrocyte lysis in an ammonium chloride buffer, the cells were passed through a 40-µm strainer and counted. The single-cell suspension was then washed twice in complete RPMI 1640 media, and, finally, 40,000 cells/ml were seeded on non-tissue culture-treated plastic plates in DC media: complete RPMI 1640 media supplemented with murine Granulocyte-Macrophage Colony Stimulating Factor (GM-CSF) (20 ng/ml) (PeproTech). On day 3, more DC media were added, doubling the volume in each plate, and, on day 6, half of the DC media was removed and replaced with fresh DC media. On day 9, half of the media was refreshed and dexamethasone (final concentration of 1 µM; D8893, Merck) was added only to the cells differentiating to tolDCs. On day 12, the whole volume was collected and centrifuged, and Lipopolysaccharide (LPS) (100 ng/ml) (*Escherichia coli* O55:B5 Lipopolysaccharides; Merck) was added to mDC and tolDC plates in DC media for 20 h. On day 13, imDCs, mDCs, and tolDCs were harvested, analyzed phenotypically by flow cytometry, and used in co-culture experiments. More than 90% CD11c^+^ cells were obtained in each culture as a measure of the dendritic cell purity (a scheme of this protocol is summarized in **Figure S6A**).

### Co-Culture Experiments

On day 13, imDCs, mDCs, and tolDCs from MRL/MPJ, MRL/lpr, and C57BL/6 mice were collected in PBS + EDTA (5 mM) and washed three times with RPMI 1640. After washing, the DCs were recovered and counted, and 2 × 10^4^ cells were seeded on 96-well round bottom plates in DC media. In parallel, splenocytes from the corresponding mouse strains were filtered, washed, and counted after erythrocyte lysis. Then, 2 × 10^5^ splenocytes were seeded to co-culture them with the DCs in DC media for 3 days. In the case of C57BL/6 (DCs)–OT-I experiments, OVA (OVA A5503, Merck) was added to the co-culture (50 µg/ml), and for CD3 and CD28 stimulation, anti-CD3e (145-2C11) and anti-CD28 were used (37.51, BD Biosciences). OT-I proliferation induced with OVA by imDCs, mDCs, and tolDCs was analyzed by Carboxyfluorescein succinimidyl ester (CFSE) (Cat. No. C34554, Invitrogen) according to the manufacturer’s recommendations. Finally, cells were stained and prepared for flow cytometry.

### Statistical Analysis

Ordinary one-way ANOVA, RM (repeated measures) one-way ANOVA, two-way ANOVA, or a paired Student’s t-test was applied using Prism 9 software (GraphPad Software).

## Results

### MPJ Mice Develop a Less Severe Autoimmune Phenotype Than LPR Mice

Because of the *lpr* mutation in the *Fas* gene, for many years, LPR mice have been used to study lupus because they reproduce most of the classic and severe symptoms observed in patients with SLE in the short term (3–4 months): glomerulonephritis, circulating antibodies against nuclear antigens, central nervous system inflammation, skin rash, and an aberrant accumulation of inflammatory CD4 and DN T cells (33). However, little is known about evolution of newly reported T-cell populations that may be important in the pathology that develops in the control MPJ mouse strain that does not carry the *lpr* mutation but that exhibits a mild form of lupus relative to the LPR mice in aged animals (>5 months).

**Figure 1:**
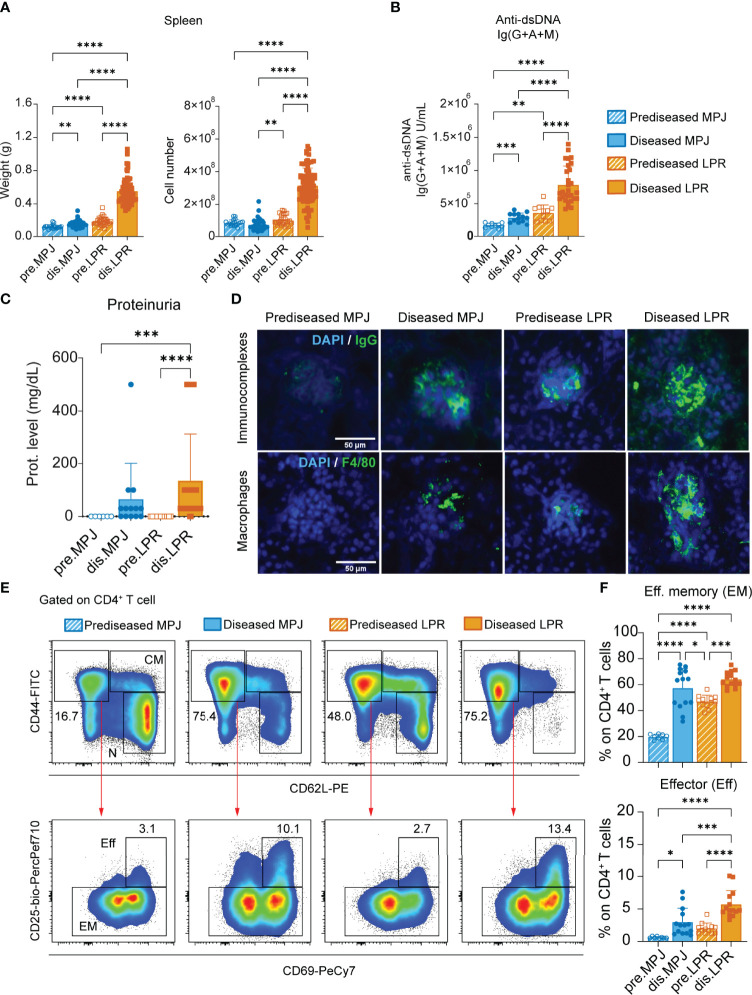
Characterization of MPJ and LPR mice differentiating two lupus disease states. **(A)** Spleen weight (left) and cell number (right). **(B)** Levels of Ig(G+A+M) antibodies against double-stranded DNA (dsDNA) determined by ELISA. **(C)** Protein concentration in urine (0, 30, 100, and 500 mg/dl) determined using urine dipsticks. **(D)** Representative immunofluorescence images from frozen kidney sections (4–10 µm) stained with DAPI (4′,6-diamidino-2-phenylindole) (blue), goat anti-mouse IgG (green) for immunocomplexes (top), and a primary rat anti-mouse F4/80 with secondary goat anti-rat IgG (green) for macrophages (bottom). Scale bar is 50 µm. **(E)** Representative flow cytometry plots gated on splenic CD4^+^ T cells (CD3^+^B220^−^CD8^−^CD4^+^) showing effector (Eff), effector memory (EM), central memory (CM), and naïve (N) gates. **(F)** Proportion of effector (Eff: CD44^+^CD62L^−^CD25^+^CD69^+^) and effector memory (EM: CD44^+^CD62L^−^CD25^−^) phenotypes among splenic CD4^+^ T cells. The data represent the mean ± SD: *P < 0.05; **P < 0.01; ***P < 0.001; ****P < 0.0001 by one-way ANOVA with a Tukey multi-comparison post-test. Each point represents a single animal: prediseased MPJ (pre.MPJ), n = 20 **(A)** 10 **(B)** 6 **(C)** and 8 **(E, F)** diseased MPJ (dis.MPJ), n = 40 **(A)** 13 **(B)** 13 **(C)** and 14 **(E, F)**; prediseased LPR (pre.LPR), n = 31 **(A)** 11 **(B)** 12 **(C)** and 12 **(E , F)** diseased LPR (dis.LPR), n = 74 **(A)** 29 **(B)** 38 **(C)** and 14 **(E, F)**.

We first classified the MPJ and LPR mice as predisease and diseased mice according to the different parameters associated with lupus progression described previously (**Table 1**) (34). Diseased LPR mice displayed intense splenomegaly with an exacerbated number of cells, whereas only a subtle increase in spleen weight was detected in diseased MPJ mice (**Figure 1A**). We also measured total anti–double-stranded DNA (dsDNA) Ig(G+A+M) in blood serum, a canonical clinical sign that is strongly correlated with disease status (35). Diseased MPJ and LPR mice had elevated levels of anti-dsDNA antibodies relative to their prediseased counterparts (**Figure 1B**). As these antibodies could contribute to lupus glomerulonephritis through their accumulation in the kidneys, we also analyzed impaired renal function and glomerular histology. Indeed, proteinuria (**Figure 1C**), immune complex deposition, and macrophage infiltration (**Figure 1D**) were evident in diseased LPR and MPJ mice. At the cellular level, the proportions of different phenotypes (36) of splenic CD4^+^ and CD8^+^ T cells (**Figure S1A**) and the proportion of DN and DN B220^+^ T cells (**Figures S1B, C**) were analyzed by flow cytometry (see gating strategy in **Figure S1D**). A higher percent of effector (CD44^+^CD62L^−^CD25^+^CD69^+^) and effector memory (CD44^+^CD62L^−^CD25^−^) CD4^+^ T cells was detected in diseased MPJ and LPR mice (**Figures 1E** **, F**). However, the characteristic accumulation of DN T cells was only observed in LPR animals (**Figures S1B, C**). Globally, diseased animals showed alterations in all the classic parameters studied, yet diseased MPJ mice exhibited a less severe pathology compared to diseased LPR mice.

**Table 1:** Summary of the parameters analyzed in the MRL/MPJ (MPJ) and MRL/lpr (LPR) animals classified as prediseased and diseased.

							Kidney		
	Spleen (g)	Age (weeks)	Anti-dsDNAIg (U/ml)	Eff. Memory (% CD4^+^ T cell)	DN B220^+^ (%Total viable cells)	Skin lesions	IgG	F4/80	Proteinuria
Prediseased MPJ	<0.3	<12	<2 × 10^5^	<20	<1	No	—	—	—
Diseased MPJ	<0.3	>22	>2 × 10^5^	>20	<1	Yes	++	++	++
Prediseased LPR	<0.3	<12	<4 × 10^5^	<60	<10	No	++	++	—
Diseased LPR	>0.3	>12	>4 × 10^5^	>60	>10	Yes	+++	+++	+++

Regarding renal function and kidney histology, (—): no manifestation; (++): mild manifestation; and (+++) severe manifestation.

### Helios Expression Is Associated With the CD4^+^ Tconv and CD4^+^ Treg Effector Phenotype in MPJ and LPR Mice

**Figure 2:**
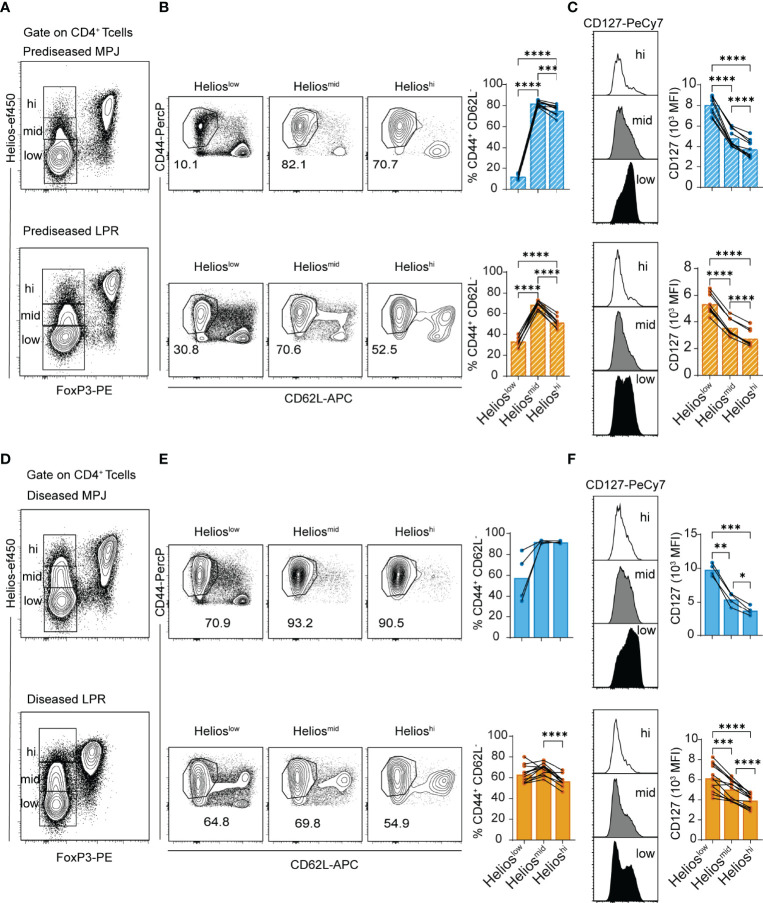
Helios expression and effector phenotype of CD4^+^ Tconvs in MPJ and LPR mice. **(A)** Representative flow cytometry plots pregated on splenic CD4^+^ T cells (CD3^+^CD4^+^) from prediseased MPJ (top) and LPR (bottom) mice, in which three different CD4^+^ Tconv (FoxP3^−^) populations are indicated through their Helios expression (Helios^low/mid/hi^). **(B)** Representative effector phenotype (CD44^+^CD62L^−^) plots of Helios^low^, Helios^mid^ and Helios^hi^ CD4^+^ Tconv cells, and their percentages (right) relative to prediseased MPJ and LPR mice. **(C)** Representative CD127 histograms gated on CD4^+^ Tconv Helios^low^, Helios^mid^, or Helios^hi^ (left), and their MFI quantification (right) in prediseased MPJ and LPR mice. **(D)** Same as in **(A)** but from diseased mice. **(E)** Same as in **(B)** but from diseased mice. **(F)** Same as in **(C)** but from diseased mice. The data represent the mean ± SD: *P < 0.05; **P < 0.01; ***P < 0.001; ****P < 0.0001 by RM one-way ANOVA with a Tukey multi-comparison post-test. Each point represents a single animal: prediseased MPJ (pre.MPJ), n = 10 **(A–C)** diseased MPJ (dis.MPJ), n = 4 **(A–C)** prediseased LPR (pre.LPR), n = 9 **(D–F)** and diseased LPR (dis.LPR), n = 12 **(D–F)**.

Once we had performed the general analysis to distinguish the disease states in the MPJ and LPR mice, we continued to evaluate the spleen in more detail, assessing Helios as a molecular marker of the CD4^+^ T-cell compartment as it has generated significant interest as a proposed marker of disease progression, particularly in rheumatoid diseases like SLE (37). We first identified three phenotypically different populations of Tconv CD4^+^ T cells (CD3^+^CD4^+^FoxP3^−^) in terms of their Helios signal intensity. Helios expression by CD4^+^ Tregs was established as a biological standard of strong expression (Helios^hi^), with the Helios levels in CD4^+^ Tconvs serving as a measure of weak expression (Helios^low^) and intermediate Helios expression (Helios^mid^) defined as the levels between these (see gating strategy in **Figure S2A**). Diseased MPJ and LPR animals all showed elevated proportions of Helios^mid/hi^ CD4^+^ Tconv cells relative to the prediseased MPJ mice (**Figures S2B, C**). Consequently, a phenotypic analysis of canonical T-cell activation markers (ICOS, CD127, CD44, and CD62L) was carried out by flow cytometry. As a result, Helios^mid/hi^ CD4^+^ Tconvs had an enhanced effector phenotype (CD44^+^CD62L^−^) relative to Helios^low^ CD4^+^ Tconvs in prediseased LPR and particularly in MPJ mice (**Figures 2A** **, B**). However, no significant difference was observed in CD4^+^ Tconvs from the diseased counterparts of these mice, probably because these cells are already maximally activated (**Figures 2D** **, E**). Interestingly, Helios^mid^ CD4^+^ Tconvs from prediseased animals seemed to present a stronger effector phenotype than Helios^hi^ CD4^+^ Tconvs (**Figure 2B**). Nonetheless, there was a constant correlation between elevated Helios expression and CD127 downregulation in CD4^+^ Tconvs in all animals (**Figures 2C** **, F**).

**Figure 3:**
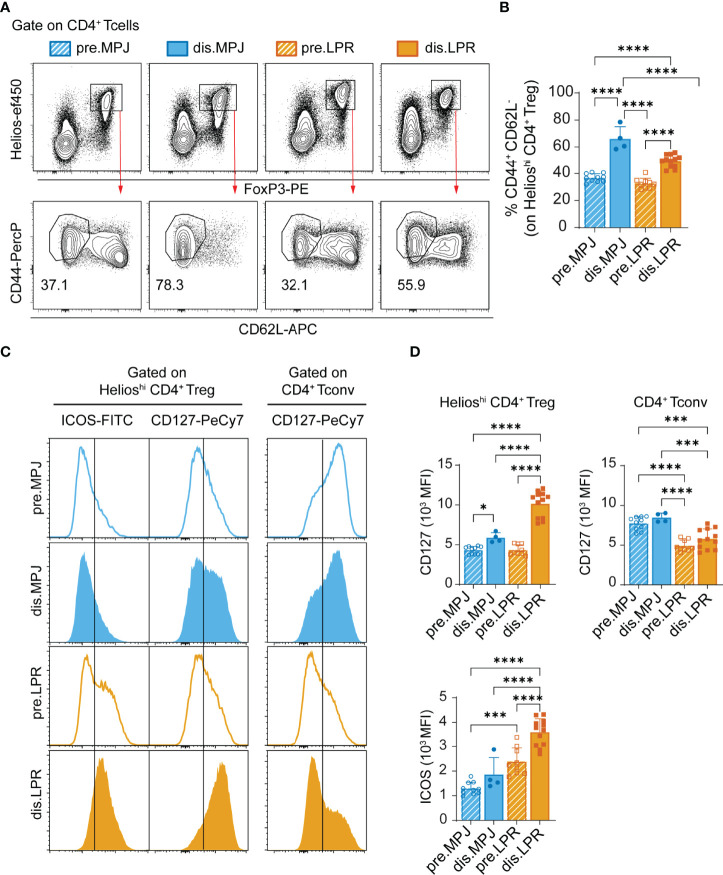
Helios expression and effector phenotype of CD4^+^ Tregs in MPJ and LPR mice. **(A)** Representative flow cytometry plots pregated on splenic CD4^+^ T cells (CD3^+^CD4^+^), in which Treg (FoxP3^+^Helios^hi^, top) and their effector (CD44^+^CD62L^−^) populations (bottom) are indicated. **(B)** Percentage of splenic CD4^+^ Treg cells with an effector phenotype. **(C)** Representative ICOS and CD127 histograms gated on CD4^+^ Treg (left) and CD4^+^ Tconv (right). **(D)** MFI quantification of (**C**). The data represent the mean ± SD: *P < 0.05; ***P < 0.001; ****;P < 0.0001 by one-way ANOVA with a Tukey multi-comparison post-test. Each point represents a single animal: prediseased MPJ (pre.MPJ), n = 10 **(A–D)** diseased MPJ (dis.MPJ), n = 4 **(A–D)** prediseased LPR (pre.LPR), n = 9 **(A–D)** and diseased LPR (dis.LPR), n = 12 **(A–D)**.

In terms of CD4^+^ Tregs (CD3^+^CD4^+^FoxP3^+^Helios^hi^), diseased MPJ and LPR mice also had a higher proportion of effector cells in the spleen (**Figures 3A** **, B**), and the ICOS and CD127 surface receptors were upregulated, the latter in contrast to the expression seen in the CD4^+^ Tconvs (**Figures 3C** **, D**).

Because Helios is also present on activated CD4^+^ Tconvs (38) that are exposed chronically to cognate antigens (39, 40), our data suggest that a chronic autoimmune context is associated with the maintenance of the Helios^mid^ phenotype over time in a population of activated effector CD4^+^ T cells in these mouse models of lupus.

### Distinct Helios Expression by CD4^+^ and CD8^+^ Tregs During the Pathological Stages of MPJ and LPR Mice

**Figure 4:**
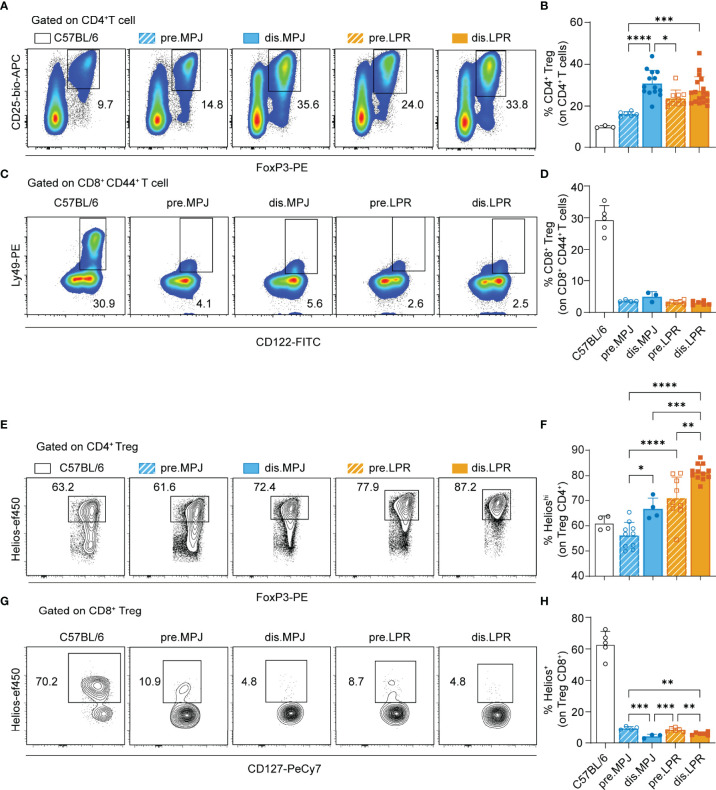
Helios expression in CD4^+^ and CD8^+^ Tregs from MPJ and LPR mice. **(A)** Representative flow cytometry plots pregated on splenic CD4^+^ T cells (CD3^+^CD8^−^CD4^+^). The CD4^+^ Treg (CD25^+^FoxP3^+^) gates are indicated. **(B)** Percentage of CD4^+^ Tregs among the CD4^+^ T cells. **(C)** Representative flow cytometry plots pregated on splenic CD8^+^ CD44^+^ T cells (CD3^+^CD8^+^CD44^+^), in which the CD8^+^ Treg (CD122^+^Ly49^+^) gates are indicated. **(D)** Percentage of CD8^+^ Treg among CD8^+^ CD44^+^ T cells. **(E)** Representative flow cytometry plots pregated on CD4^+^ Tregs with the Helios^hi^ gate indicated. **(F)** Percentage of Helios^hi^ cells among CD4^+^ Tregs. **(G)** Representative flow cytometry plots pregated on CD8^+^ CD127^+^ Tregs, with the Helios^+^ gate indicated. **(H)** Percentage of Helios^+^ cells among CD8^+^ Tregs. The data represent the mean ± SD: *P < 0.05, **P < 0.01; ***,P < 0.001; ****,P < 0.0001 for comparisons between MRL animals. One-way ANOVA with a Tukey multi-comparison post-test was used. Each point represents a single animal: C57BL/6, n = 3 **(A, B)**, 5 **(C, D)**, 4 **(E, F)**, and 5 **(G, H)**; prediseased MPJ (pre.MPJ), n = 6 **(A, B)**, 4 **(C, D)**, 10 **(E, F)**, and 4 **(G, H)**; diseased MPJ (dis.MPJ), n = 14 **(A, B)**, 3 **(C, D)**, 4 **(E, F)**, and 3 **(G, H)**; prediseased LPR (pre.LPR), n = 10 **(A, B)**, 4 **(C, D)**, 9 **(E, F)**, and 4 **(G, H)**; and diseased LPR (dis.LPR), n = 18 **(A, B)**, 6 **(C, D)**, 12 **(E, F)**, and 6 **(G, H)**.

Because Helios expression in the CD4^+^ Tconv cell compartment is associated with disease progression, we wondered whether this enhanced expression is also found in other splenic immune cell populations that express Helios as a specific marker, such as CD4^+^ Tregs (see gating strategy on **Figure S3A**) and CD8^+^ Tregs (CD44^+^CD122^+^Ly49^+^; see gating strategy in **Figure S3B**). Because we used Helios in our previous definition of CD4^+^ Treg cells, we examined a new panel of markers commonly associated with these cells (CD3^+^CD8^−^CD4^+^CD25^+^FoxP3^+^). To ensure a bona fide detection of Ly49, we validated the antibody anti-Ly49C/I/F/H (clone 14B11) in MRL genetic background (**Figures S7B, C**). Furthermore, we also included an additional mouse strain as a reference: the C57BL/6 to address these populations in mice without genetic susceptibility to lupus. Unexpectedly, whereas the proportion of CD4^+^ Tregs increased in MPJ and LPR mice in the disease state (**Figures 4A** **, B**), CD8^+^ Tregs were almost completely absent from all MPJ and LPR animals, independent their pathological state and relative to C57BL/6 (**Figures 4C** **, D**). Although, absolute splenic numbers of CD8^+^CD44^+^CD122^+^ T cells were increased in diseased LPR mice because of an exacerbated splenomegaly (**Figures S4A, B**). Similar behavior was evident in terms of Helios expression, and, whereas Helios expression was enhanced in CD4^+^ Tregs from diseased MPJ and LPR mice (**Figures 4E** **, F**), its expression was dramatically reduced in CD8^+^ Tregs from these mice relative to the C57BL/6 mice (**Figures 4G** **, H**). In this regard, there was still a mild yet significant difference between prediseased and diseased MPJ and LPR mice, the latter presenting the weakest Helios expression (**Figures 4G** **, H**). In terms of the global spleen percentages and Helios expression, data showed a contrasting behavior between CD4^+^ Tregs and CD8^+^ Tregs from MPJ and LPR mice relative to the C57BL/6 mice, even in the prediseased animals.

### The CD8^mid^ CD44^+^ Population Does Not Present a Treg Phenotype in Prediseased MPJ and LPR Mice

**Figure 5:**
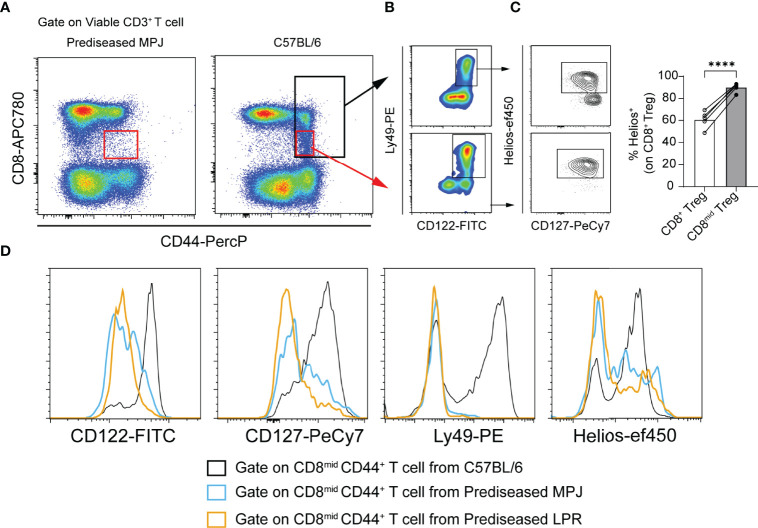
Phenotypic analysis of CD8^mid^ CD44^+^ populations in prediseased MPJ and LPR mice. **(A)** Representative flow cytometry plots pregated on viable CD3^+^ T cell from prediseased MPJ (left) and C57BL/6 mice (right). **(B)** Representative flow cytometry plots of CD8^+^CD44^+^ and CD8^mid^CD44^+^ gates from C57BL/6 mice and their Treg phenotype. **(C)** Percentage of Helios^+^ cells among CD8^+^ and CD8^mid^ Tregs. The data represent the mean values. **(D)** Representative histograms pregated on CD8^mid^CD44^+^ T cells from C57BL/6 (black), prediseased MPJ (blue), and prediseased LPR mice (orange): ****P < 0.0001 by a paired Student t-test. Each point represents a single animal (C57BL/6, n = 5).

Apart from its specific expression by Tregs, Helios has also been proposed as a marker of CD8 T cells that undergo CD8 downregulation after encountering antigens in unmanipulated naïve mice under pathogen-free conditions (41, 42). As a result, some proinflammatory DN T cells with an effector phenotype (CD127^low^) can be generated. Given that our experiments were performed in pathogen-free conditions, it would be reasonable to consider that the immune system of all the animals examined were only exposed to microorganisms from their diet and self-antigens. Indeed, Helios was particularly enriched in a small fraction of activated CD8^mid^ CD44^+^ T cells, with a clear Treg phenotype (CD122^+^Ly49^+^CD127^+^Helios^+^) only evident in naïve C57BL/6 mice (**Figures 5A** **–C**). By contrast, the equivalent population in prediseased MPJ and LPR animals exhibited a non-Treg effector phenotype, with lower levels of CD122, CD127, Ly49, and Helios (**Figure 5D**). Hence, T cells undergoing CD8 downregulation from MRL animals could be more prone to adopt a non-Treg effector phenotype than equivalent cells from C57BL/6 mice.

### Reduced Helios Expression in Different T-Cell Subsets From Diseased MPJ and LPR Mice

**Figure 6:**
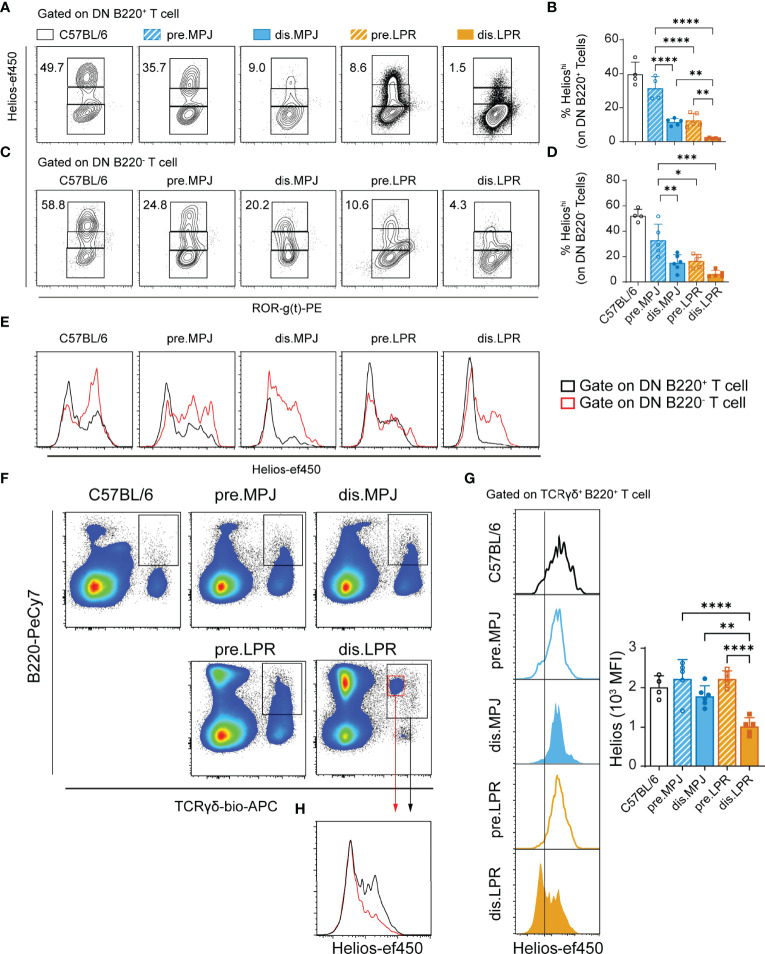
Helios levels in different T-cell subsets from MPJ and LPR mice. **(A)** Representative flow cytometry plots pregated on splenic DN B220^+^ T cells (CD3^+^TCRγδ^−^B220^+^CD4^−^CD8^−^), in which three different gates are indicated based on Helios expression (Helios^low/mid/hi^). **(B)** Helios^hi^ frequencies on DN B220^+^ T cells. **(C)** Representative flow cytometry plots pregated on splenic DN B220^−^ T cells (CD3^+^TCRγδ^−^B220^−^CD4^−^CD8^−^). **(D)** Helios^hi^ frequencies among DN B220^−^ T cells. **(E)** Representative histograms of Helios in DN T-cell populations: DN B220^+^ (black) and DN B220^−^ (red). **(F)** Representative flow cytometry plots pregated on splenic CD3^+^ T cells, indicating the TCRγδ^+^ B220^+^ gates. **(G)** Representative Helios histograms gated on TCRγδ^+^ B220^+^ (left) and their quantification (MFI: right). **(H)** Representative Helios histograms comparing two distinct TCRγδ^+^ B220^+^ subsets (red and black) from diseased LPR mice. The data represent the mean ± SD: *P < 0.05, **P < 0.01, ***P < 0.001, ****P < 0.0001 for comparisons between MRL animals. One-way ANOVA with a Tukey multi-comparison post-test. Each point represents a single animal: prediseased MPJ (pre.MPJ), n = 5 **(A–D, G)**; diseased MPJ (dis.MPJ), n = 6 **(A–D, G)**; prediseased LPR (pre.LPR), n = 5 **(A–D, G)**; and diseased LPR (dis.LPR), n = 5 **(A–D, G)**.

Apart from the CD8^+^ and CD4^+^ T cells, there are other T-cell populations in both human and mouse SLE that fulfill a relevant role in the immune pathology of this disease, such as DN T (4, 11) and γδ T cells (43). As such, we explored the Helios expression in these DN (CD3^+^TCRγδ^−^CD4^−^CD8^−^) and γδ (CD3^+^TCRγδ^+^) T cells, studying them in MPJ, LPR, and C57BL/6 mice. We divided Helios expression into the three different categories indicated previously (Helios^low/mid/hi^, see **Figure S2A**), based on the levels in CD4^+^ T cells as internal biological controls for each mouse (see gating strategy in **Figure S5A**). Because B220 is a characteristic marker expressed by DN T cells from LPR mice, we separately analyzed DN B220^+^ and DN B220^−^ T cells. In this regard, we observed a global reduction in the proportion of Helios^hi^ DN B220^+^ and DN B220^−^ T cells in diseased MPJ and LPR animals, in accordance with the results from CD8^+^ Tregs (**Figures 6A** **–D**). However, interestingly, Helios expression in DN B220^−^ T cells was consistently higher than in their DN B220^+^ counterparts in all the animals tested (**Figure 6E**). The strongest Helios expression was also detected in both the B220^+/−^ DN T-cell populations from C57BL/6 mice. By contrast, there were more TCRγδ^+^ B220^+^ T cells in all the MPJ and LPR mice than in the C57BL/6 mice (**Figure S5B**), most of which presented a Helios^mid^ phenotype and no RORγ(t) expression, in contrast to their TCRγδ^+^ B220^−^ counterparts (**Figure S5C**). However, in diseased LPR mice, there was a significant reduction in Helios in TCRγδ^+^ B220^+^ cells (**Figures 6F** **, G**), a cell subset that is particularly enriched in those animals (**Figure 6H**). Together, these data reinforce the idea that there is a distinct pattern of Helios expression in different splenic T-cell subpopulations and that Helios downregulation in these cells is associated with more severe forms of lupus, irrespective of the increased levels of this factor in the CD4^+^ T-cell compartment.

### Helios Expression in CD8^+^ T Cells Is Promoted *In Vitro* by DCs in C57BL/6 Mice

Having analyzed splenic Helios expression in different T-cell populations and in distinct states of lupus, we evaluated, specifically in CD8^+^ T cells, the influence of cellular context in modulating its expression. Given that antigenic presentation by functionally and phenotypically different types of DCs would provide distinct stimuli, we performed some classical co-culture experiments with DCs and CD8^+^ T cells. To address that, we generated *in vitro* conventional imDCs, mDCs, and tolDCs from C57BL/6 mice using a slightly modified version of a bone marrow–derived DC protocol described previously (32) (scheme in **Figure S6A**). After 13 days in culture, DCs (>90% CD11c^+^ cells; **Figure 6B**) from C57BL/6 mice were harvested, washed, and cultured with spleen cells from OT-I animals in a 1:10 (DC:splenocyte) ratio for 3 days.

**Figure 7:**
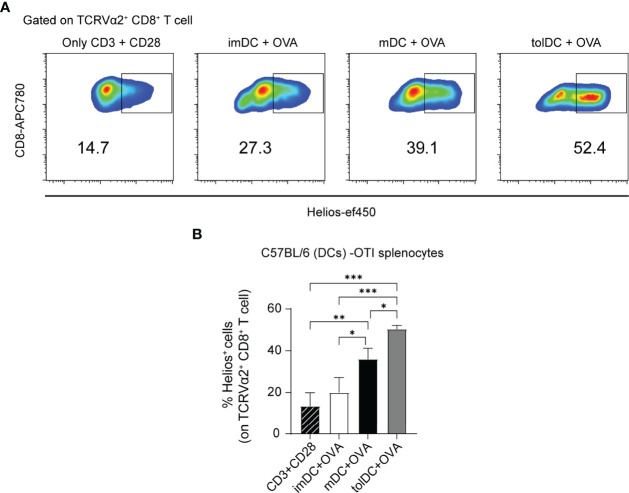
Helios expression in CD8^+^ T cells from OT-I mice co-cultured with different types of DCs. **(A)** Representative flow cytometry plots gated on TCRVα2^+^ CD8^+^ showing Helios expression in a 3-day co-culture experiment. Bone marrow dendritic cells (DCs): immature (imDC), mature (mDC), and tolerogenic (tolDC) from C57BL/6 were co-cultured with splenocytes from OT-I mice in presence of OVA. As a positive control of stimulation, anti-CD3 and anti-CD28 antibodies were used. **(B)** Percentage of Helios^+^ cells among TCRVα2^+^ CD8^+^ T cells. The data represent the mean ± SD: *P < 0.05; **P < 0.01; ***P < 0.001 by one-way ANOVA with a Tukey multi-comparison post-test (n = 2–4 independent experiments).

We first performed a classic presentation-activation assay to evaluate the different DC activities by co-culturing them with splenocytes from OT-I mice, whose CD8^+^ T cells exhibit a transgenic TCR that primarily recognizes OVA_257–264_ when presented by MHC class I molecules. Using the CFSE dilution method, we measured the ability of OVA-incubated imDC, mDC, and tolDC cells from C57BL/6 mice to induce OT-I cell proliferation. As a positive control of proliferation, we used anti-CD3 and anti-CD28 stimulation. As expected, while mDCs exhibited the greatest OT-I cell proliferation (on TCR Vα2^+^CD8^+^ cells), tolDCs displayed the weakest proliferation and imDCs an intermediate response (**Figure S6C**). However, interestingly, in these C57BL/6 (DC)–OT-I (splenocyte) experiments, the highest Helios upregulation on TCR Vα2^+^CD8^+^ T cells was seen in presence of tolDCs, although mDC culture also provoked increased Helios expression relative to the response to anti-CD3 and anti-CD28 stimulation (**Figures 7A** **, B**). Alternatively, without OVA, mDCs were not able to induce OT-I cell proliferation and Helios levels remained low (**Figures S6D, E**). Overall, these *in vitro* data indicate that Helios expression on CD8^+^ T cells is facilitated by dendritic stimuli and, specifically, that the greatest Helios induction was reached when CD8^+^ T cells were co-cultured with tolDCs.

### Helios Expression in CD8^+^ T Cells Is Not Induced by DCs in MRL Animals

**Figure 8:**
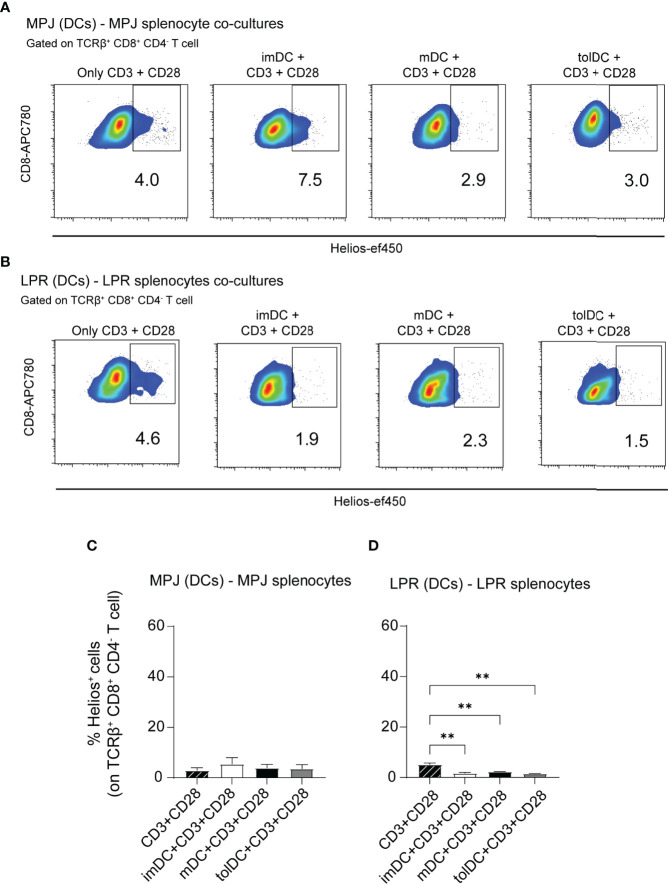
Helios expression in CD8^+^ T cells from MRL mice co-cultured with different types of DCs. **(A)** Representative flow cytometry plots gated on TCRβ^+^CD8^+^CD4^−^ T cells showing Helios expression in a 3-day co-culture experiment with bone marrow derived dendritic cells (DCs) and splenocytes from MPJ mice in the presence of CD3 and CD28 stimuli. **(B)** Representative flow cytometry plots gated on TCRβ^+^CD8^+^CD4^−^ T cells showing Helios expression in 3-day co-culture experiments with bone marrow–derived DCs and splenocytes from LPR mice, in the presence of CD3 and CD28 stimuli. **(C)** Percentage of Helios^+^ cells among MPJ TCRβ^+^CD8^+^CD4^−^ T cells. **(D)** Percentage of Helios^+^ cells among LPR TCRβ^+^CD8^+^CD4^−^ T cells. The data represent the mean ± SD: **P < 0.01 by one-way ANOVA with a Tukey multi-comparison post-test (n = 2–4 independent experiments).

To address whether Helios expression in CD8^+^ T cells could be facilitated by DCs in MRL animals as well, we performed similar co-culture experiments as previously described. However, given that there are no specific antigens to be presented (in contrast to OVA presentation system), for these experiments, DCs were cultivated with splenocytes in presence of an anti-CD3 and anti-CD28 stimulation in all cases. In the MPJ (DC)–MPJ (splenocyte) experiments, there were no significant differences in terms of Helios expression in CD8^+^ T cells (TCRβ^+^CD8^+^CD4^−^) when tested with DCs and artificial stimuli or with artificial stimuli alone (**Figures 8A** **, C**). However, although Helios expression by MPJ and LPR splenocytes exposed to artificial stimuli was similar, the presence of LPR DCs downregulated Helios in LPR CD8^+^ T cells (**Figures 8B** **, D**).

In contrast to C57BL/6 experiments, these results suggest that Helios upregulation in CD8^+^ T cells from MRL animals does not occur in presence of dendritic stimuli. In that context, interestingly, Helios expression was diminished in CD8^+^ T cells co-cutured specifically with LPR DCs when compared with cells exposed to artificial stimuli alone.

### Altered Phenotype of DCs From Diseased LPR Mice *In Vitro* and *In Vivo*

**Figure 9:**
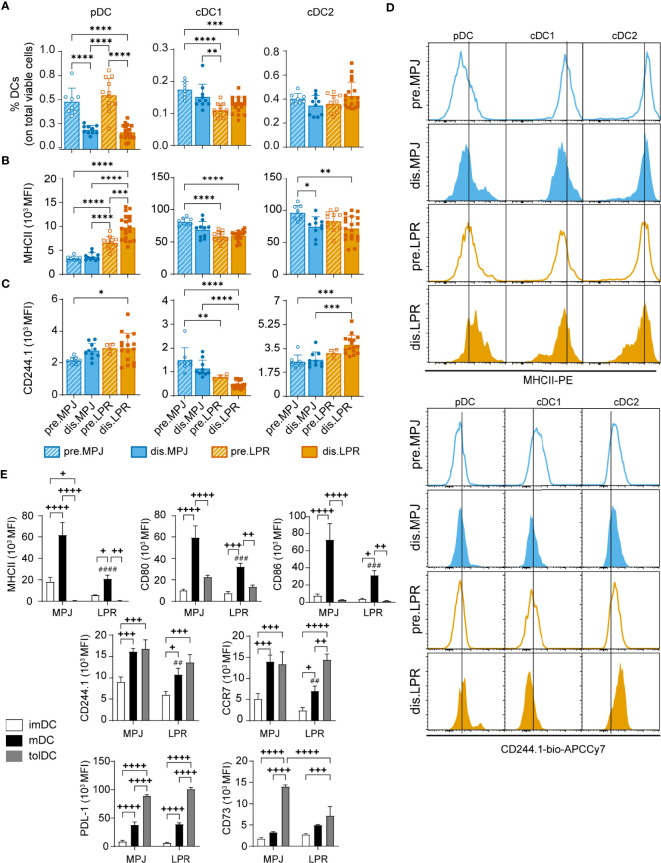
*In vitro* and *in vivo* phenotypic analysis of DCs from MPJ and LPR mice. **(A)** Percentages of splenic plasmacytoid (pDC: CD11c^low^CD11b^-^B220^+^PDCA-1^+^CD3/CD19^−^, left), conventional type 1 (cDC1: CD11c^hi^CD11b^−^CD8α^+^B220^−^PDCA-1^−^CD3/CD19^−^, middle), and conventional type 2 (cDC2: CD11c^hi^CD11b^+^CD8α^−^B220^−^PDCA-1^−^CD3/CD19^−^, right) dendritic cells (DCs) among the total viable cells from MPJ and LPR mice. **(B)** Quantification (MFI) of the MHCII expression on pDCs (left), cDC1 (middle), and cDC2 (right). **(C)** Quantification (MFI) of the CD244.1 expression on pDCs (left), cDC1 (middle), and cDC2 (right). **(D)** Representative histograms of **(B)** (top) and **(C)** (bottom). The data represent the mean ± SD: *P < 0.05; **P < 0.01; ***P < 0.001; ****P < 0.0001 by one-way ANOVA with a Tukey multi-comparison post-test. Each point represents a single animal: prediseased MPJ (pre.MPJ), n = 8 **(A–C)**; diseased MPJ (dis.MPJ), n = 10 **(A–C)**; prediseased LPR (pre.LPR), n = 4–12 **(A–C)**; and diseased LPR (dis.LPR), n = 16 **(A–C)**. **(E)** Quantification (MFI) of different DC markers on immature (imDC), mature (mDC), and tolerogenic (tolDC) DCs from the bone marrow of MPJ and LPR mice. The data represent the mean ± SD: ^+^P < 0.05, ^++^P < 0.01, ^+++^P < 0.001, and ^++++^P < 0.0001 for comparisons between imDCs, mDCs, and tolDCs from MPJ or LPR animals; and ^##^P < 0.01, ^###^P < 0.001, and ^####^P < 0.0001 for the comparisons between mDCs from MPJ and LPR animals, using a two-way ANOVA with a Tukey multi-comparison post-test.

Finally, in our *in vitro* experiments, because we observed that dendritic context could influence Helios expression in CD8^+^ T cells, we decided to study the phenotype and proportions of different DC subsets in MPJ and LPR animals. In more detail, we first analysed *in vivo* the proportions and the MHCII and CD244.1 expression by flow cytometry on splenic pDCs (CD11c^low^CD11b^−^B220^+^PDCA-1^+^), cDC1 (CD11c^hi^CD11b^−^CD8α^+^), and cDC2 cells (CD11c^hi^CD11b^+^CD8α^−^) (see gating strategy in **Figure S6F**). We included CD244 because it has recently been described as a relevant receptor in DCs, with implications in SLE (44) and only the CD244.1 allotype because CD244.2 allotype does not seem to be present in MRL animals (**Figure S7A**). On the one hand, reduced proportions of pDCs were seen in diseased MPJ and LPR animals (**Figure 9A**), although the levels of MHCII and CD244.1 in these cells were higher in LPR mice than in MPJ mice (**Figures 9B** **–D**). Interestingly, there were significant differences and similarities between the two major types of conventional DCs (cDC1 and cDC2 cells). Whereas the proportions of cDC1 cells was lower and CD244.1 was expressed more weakly in diseased LPR relative to prediseased MPJ mice, cDC2 cells had higher levels of CD244.1, and there were no differences in terms of the proportions of these cells (**Figures 9A** **, C**). In both type of cDCs, MHCII downregulation was detected in diseased LPR animals (**Figure 9B**).

To explore any possible intrinsic defects in DC development, we differentiated imDCs, mDCs, and tolDCs from prediseased MPJ and LPR bone marrow, as described previously. After 13 days in culture, a phenotypic analysis of different markers of dendritic maturation (CD80, CD86, MHCII, CD244.1, and CCR7) and tolerogenic markers (CD73 and PDL-1) was performed. Regarding maturation markers, as expected mDCs presented higher levels of MHCII, CD80, CD86, CD244.1, and CCR7 in comparison to their imDC counterparts. However, consistent with the observations *in vivo*, mDCs from the LPR mice exhibited lower levels of these surface proteins than MPJ mDCs (**Figure 9E**). Conversely, tolDCs presented weaker MHCII, CD80, and CD86 expression yet increased expression of PDL-1 and CD73 compared to mDCs from both LPR and MPJ mice. Interestingly, the CD73 tolerogenic marker was significantly downregulated in LPR tolDCs relative to MPJ tolDCs (**Figure 9E**). In summary, these results suggest an intrinsic alteration in the DC compartment correlates with the severe forms of lupus in LPR mice.

## Discussion

In the present work, we described aberrant Helios expression in different T-cell subsets from MPJ and LPR mouse models of lupus relative to C57BL/6 mice that are not prone to lupus. Assessing these well-known lupus models from this new perspective, most of the data obtained indicates that diseased MPJ mice exhibit a less severe pathology than LPR mice, although both consistently present an altered DC compartment and aberrant Helios expression in CD4^+^ Tconv, CD4^+^ Treg, CD8^+^ Treg, and DN T cells as the disease develops. Notably, the most evident difference between MRL and C57BL/6 mice is that, virtually, all CD8^+^ Tregs were absent in MRL animals, expressing virtually no Helios, even in young predisease MPJ mice. Thus, we propose here a new link between the disruption of tolDCs activity and CD8^+^ Treg deficiencies in this autoimmune disease.

As one of the classic parameters examined in relation to lupus, the proportion of splenic effector memory CD4^+^ T cells has, for many years, been used to evaluate immune status in models of lupus. Although we did find significant differences between diseased MPJ and diseased LPR mice in terms of effector (CD44^+^CD62L^−^CD25^+^CD69^+^) CD4^+^ T cells, more robust differences were found by comparing CD127, ICOS, and Helios expression, specifically in CD4^+^ Tregs, revealing a stronger correlation between disease severity and the effector CD4^+^ Treg cell repertoire. Because these markers are associated with a stronger activation phenotype of mouse CD4^+^ Tregs (45), it seems that their upregulation may be a compensatory mechanism, albeit unsuccessful, to avoid enhanced autoimmunity. Although contrasting results have been reported previously in terms of the total number of CD4^+^ FoxP3^+^ Tregs in patients with SLE (46–48), elsewhere consistently high CD4^+^ FoxP3^+^ Helios^+^ numbers were correlated with disease activity (48–50). Furthermore, from a flow cytometry Helios analysis, we provide more insights about its distinct levels of expression, Helios^low/mid/hi^, beyond a mere Helios^+/−^ discrimination.

Among the different CD8^+^ Treg cell subsets defined (51, 52), we focused on CD8^+^ Tregs expressing the triad of the CD44, CD122, and Ly49 surface markers (in addition to Helios) due to their presence in steady-state naïve animals and their reported implications in the lupus-like phenotypes (12, 15), as well as in other autoimmune models (17, 53), and their very recent description in human autoimmunity as KIR^+^ CD8^+^ T cells (9). In this sense, although other CD8^+^ Treg populations have been described with important roles in cell therapy, such as CD8^+^CD103^+^ (54), CD8^+^FoxP3^+^, and CD8^+^CD28^−^ Tregs (55), most of them are essentially induced *in vitro* and barely in young unmanipulated mice, unlike CD8^+^ Tregs [CD44^+^CD122^+^Ly49^+^ (12, 52)].

Interestingly, MPJ and LPR mice had neither an evident CD8^+^ Treg population compared to naïve C57BL/6 mice nor abundant Helios expression, regardless of their pathological status and their enhanced CD4^+^ Helios^hi^ Treg compartment. Nonetheless, there was a slight but significant reduction in Helios expression by CD8^+^CD127^+^ Tregs from diseased MPJ and LPR mice relative to the predisease MPJ and LPR mice. Given the non-redundant role of CD8^+^ Tregs in controlling germinal center reactions by eliminating activated CD4^+^ T-follicular helper cells (12, 13), this intrinsic absence of CD8^+^ Treg cells could be one of the main unknown drivers of lupus in the MRL background. Indeed, other groups have reported lupus-like phenotypes after 5 months (as in MPJ) in mice with specific CD8^+^ Treg deficiencies (12, 14, 15), and, very recently, a significant reduction of Helios was described in total CD8^+^ T cells from patients with SLE (56) and in the equivalent human population to murine CD8^+^CD44^+^CD122^+^Ly49^+^ Tregs, called KIR^+^ CD8^+^ Tregs (9, 12). Together, these data suggest a pivotal role for these Helios expressing CD8^+^ Tregs in human and murine lupus, which could be further explored in future studies. In contrast, it was proposed that lupus-prone C57BL6/lpr animals presented a higher proportion of CD8^+^ Tregs and an impaired suppressive capacity due to defective killing of activated cells *via* the Fas/FasL pathway (57). However, a distinct genetic background (C57BL/6) for the *lpr* mutation was used in that work and the definition of CD8^+^ Tregs differed (CD122^+^CD49d^low^). This might reflect the importance of genetic background in immunopathologies and that better definitions for true Treg CD8^+^ cells are necessary to avoid the overlap between immune populations.

Intriguingly, the Treg phenotype (CD122^+^Ly49^+^), as well as high CD127 and Helios expression, was enriched in a CD8^mid^ CD44^+^ population found preferentially in naïve C57BL/6 mice. This is consistent with recent studies on steady-state inflammatory DN T-cell generation *via* CD8 downregulation in self-reactive CD8^+^ T cells (42, 58). This proinflammatory DN T-cell subpopulation exhibits an effector phenotype (CD127^low^) in naïve animals (41) and in lupus (59). Accordingly, and in contrast to C57BL/6 mice, the absence of the CD8^+^ Treg phenotype and the lower levels of CD127 could indicate that CD8^mid^CD44^+^ T cells from a MRL background (even predisease MPJ animals) are more prone to inflammatory DN T-cell conversion. This is also consistent with the weaker CD127 expression by Helios-deficient CD8^+^Ly49^+^ Tregs under proinflammatory conditions (16). Furthermore, inflammatory DN T cells derived from CD8^+^ T cells undergo transient Helios expression (41). As such, although specific Helios upregulation in DN B220^−^ T cells from diseased MPJ and LPR mice could indicate recent antigen exposure, lower levels of this transcription factor in their B220^+^ counterparts suggest a long-lasting presence of the T-cell repertoire and a more proinflammatory phenotype.

We also found *in vitro* that Helios was preferentially boosted in CD8^+^ T OT-I cells in the presence of tolDCs from C57BL/6 mice relative to their imDC and mDC counterparts, but not in CD8^+^ T cells co-cultured with DCs from a MRL background. Indeed, glucocorticoid induced tolDCs were also seen to elevate the CD8^+^ Treg (FoxP3^+^) numbers in the context of cancer (60) and human CD14^+^ monocytes displayed a similar behavior (61). In addition, the role of TGF-β in modulating Helios in CD8^+^ Tregs has been studied (CD44^+^CD122^+^Ly49^+^) (12), this regulatory cytokine being mainly produced by tolDCs (62). The importance of DCs in regulating the CD8^+^ Treg compartment of naïve mice is supported by the notable reduction in the CD8^+^ CD44^+^ population in a mouse model lacking DCs that also develop an autoimmune phenotype, along with splenomegaly and antinuclear antibody production (29).

Given that dendritic context seems to be important in controlling Helios on CD8^+^ T, we also analyzed phenotypically this cell compartment in MRL animals. Correlating with impaired Helios induction observed on DCs from LPR co-culture experiments, intrinsic phenotypic alterations were observed *in vivo* and *in vivo* in the DC compartment of these animals. A generalized downregulation of classic markers of dendritic maturation (MHCII, CD80, CD86, CCR7, and CD244.1) and of the tolerogenic CD73 marker could suggest a relevant role of dendritic maturation in tolerogenic function (63), thereby influencing the immune equilibrium through CD8^+^ T cells. Interestingly, CD244 KO mice present a Natural Killer (NK) cell–independent lupus-like phenotype (64), and their DCs also exhibit an aberrant proinflammatory cytokine profile, as well as less MHCII and CD86 (65). Regarding patients with SLE, CD244 was also seen to be downregulated in monocytes (66), and early studies showed reduced levels of co-stimulatory molecules on DCs and a poor stimulation of allogenic T cells (67). Future research will be needed to elucidate the specific mechanisms that drive this interaction between mature tolDCs and CD8^+^ Tregs to boost this critical regulatory subset in therapies for SLE.

In conclusion, a CD8^+^ Treg (CD44^+^CD122^+^Ly49^+^) population expressing low levels of Helios was detected in lupus-prone genetic backgrounds. Given the extended dysregulation of Helios expression to other pivotal T-cell subsets in lupus, such as CD4^+^ Tregs, Tconvs, DN, and γδ T cells, Helios expression may be a good candidate as a new and versatile marker for patients with SLE and perhaps for other autoimmune pathologies.

## Data Availability Statement

The original contributions presented in the study are included in the article/**Supplementary Material**. Further inquiries can be directed to the corresponding author.

## Ethics Statement

The animal study was reviewed and approved by CNB Ethics Committee for Animal Experimentation, CSIC Ethics Committee, and by the Division of Animal Protection of the regional government of the Comunidad de Madrid, in compliance with the national (RD 53/2013) and European Union legislation (directive 2010/637EU).

## Author Contributions

AP-M and DB designed the experiments. AP-M performed most of the experiments. AP-M and DB analyzed the data. GA assisted with some experiments and gave technical support. AP-M and DB wrote the manuscript. All authors contributed to the article and approved the submitted version.

## Funding

This work was supported by the grant PID2020-112685RB-100 funded by MCIN/AEI/10.13039/501100011033 (to DB). AP-M is a predoctoral fellow supported by a fellowship from “la Caixa” Foundation (ID 100010434, fellowship code LCF/BQ/DE18/11670013) at Molecular Biosciences Program of the Universidad Autonoma de Madrid. GA received a JAE-INTRO 2020 Fellowship from the Spanish National Research Council (CSIC, JAEINT20_EX_0049).

## Conflict of Interest

The authors declare that the research was conducted in the absence of any commercial or financial relationships that could be construed as a potential conflict of interest.

## Publisher’s Note

All claims expressed in this article are solely those of the authors and do not necessarily represent those of their affiliated organizations, or those of the publisher, the editors and the reviewers. Any product that may be evaluated in this article, or claim that may be made by its manufacturer, is not guaranteed or endorsed by the publisher.
