# Supplementary material for: Helios Expression Is Downregulated on CD8+ Treg in Two Mouse Models of Lupus During Disease Progression
Source: Front Immunol. 2022 Jun 16;13:922958. doi: 10.3389/fimmu.2022.922958 (PMC9244697; doi:10.3389/fimmu.2022.922958)
Supplement: Supplementary file 1 [file DataSheet_1.pdf]

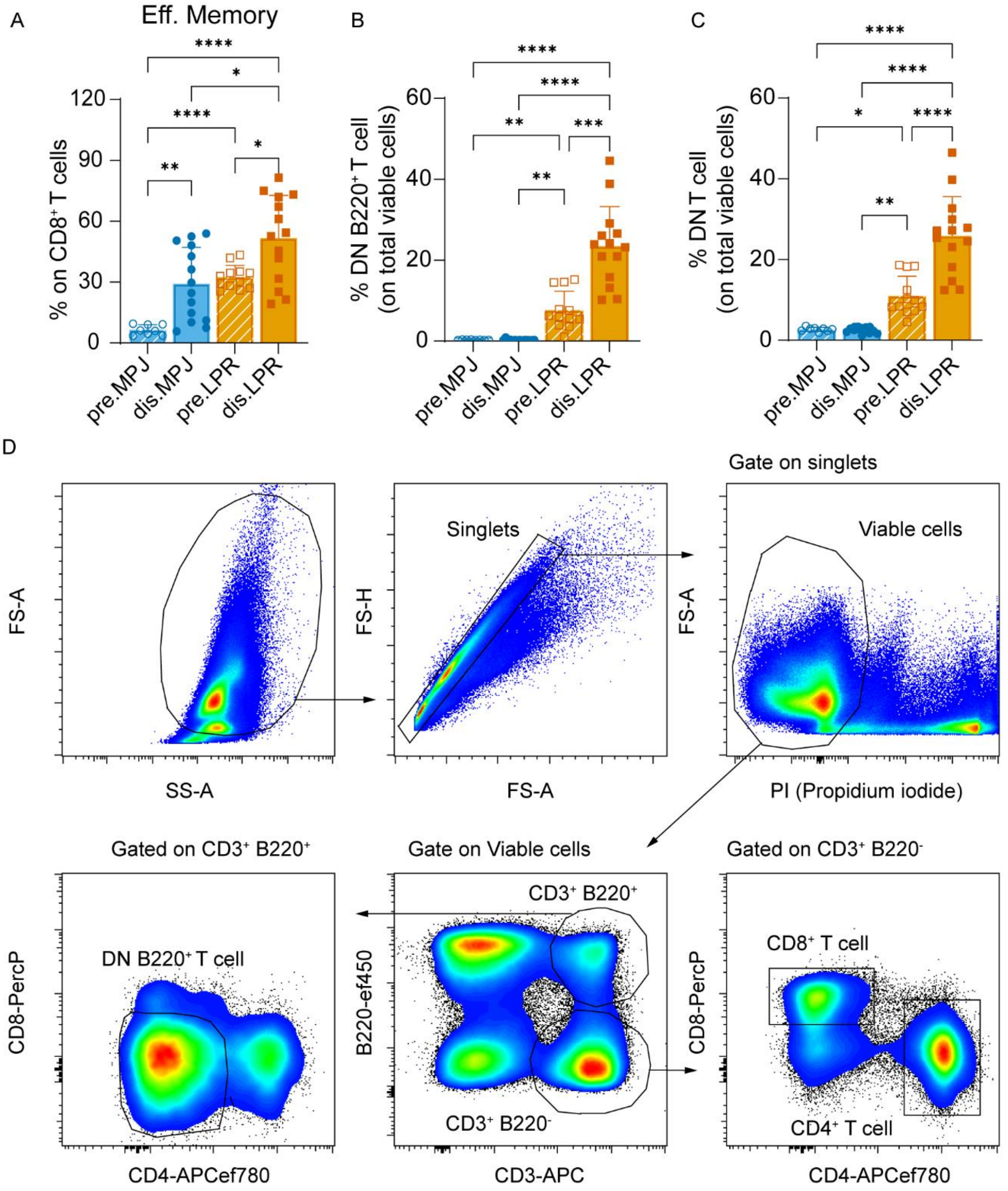


**Figure S1. Additional characterization of MPJ and LPR mice. (A)** Percentage of effector memory (EM: CD44$^{+}$CD62L$^{-}$ CD25$^{-}$) cells among splenic CD8$^{+}$ T cells (CD3$^{+}$B220$^{-}$CD8$^{+}$CD4$^{-}$). **(B)** Percentage of double negative (DN) B220$^{+}$ T cells (CD3$^{+}$B220$^{+}$CD8$^{-}$CD4$^{-}$) among the total live splenic cells. **(C)** Percentage of DN T cells (CD3$^{+}$CD8$^{-}$CD4$^{-}$) among the total live splenic cells. The data represent the mean ± SD: * $P < 0.05$; ** $P < 0.01$; *** $P < 0.001$; **** $P < 0.0001$ by one-way ANOVA with a Tukey multi-comparison post-test. Each point represents a single animal: prediseased MPJ (pre.MPJ), n = 8 (A, B and C); diseased MPJ (dis.MPJ), n = 14 (A, B and C); prediseased LPR (pre.LPR), n = 12 (A, B and C); diseased LPR (dis.LPR), n = 14 (A, B and C). **(D)** Gating strategy used for the splenic DN, CD8$^{+}$ and CD4$^{+}$ T cells. The plots are from a prediseased LPR mouse.

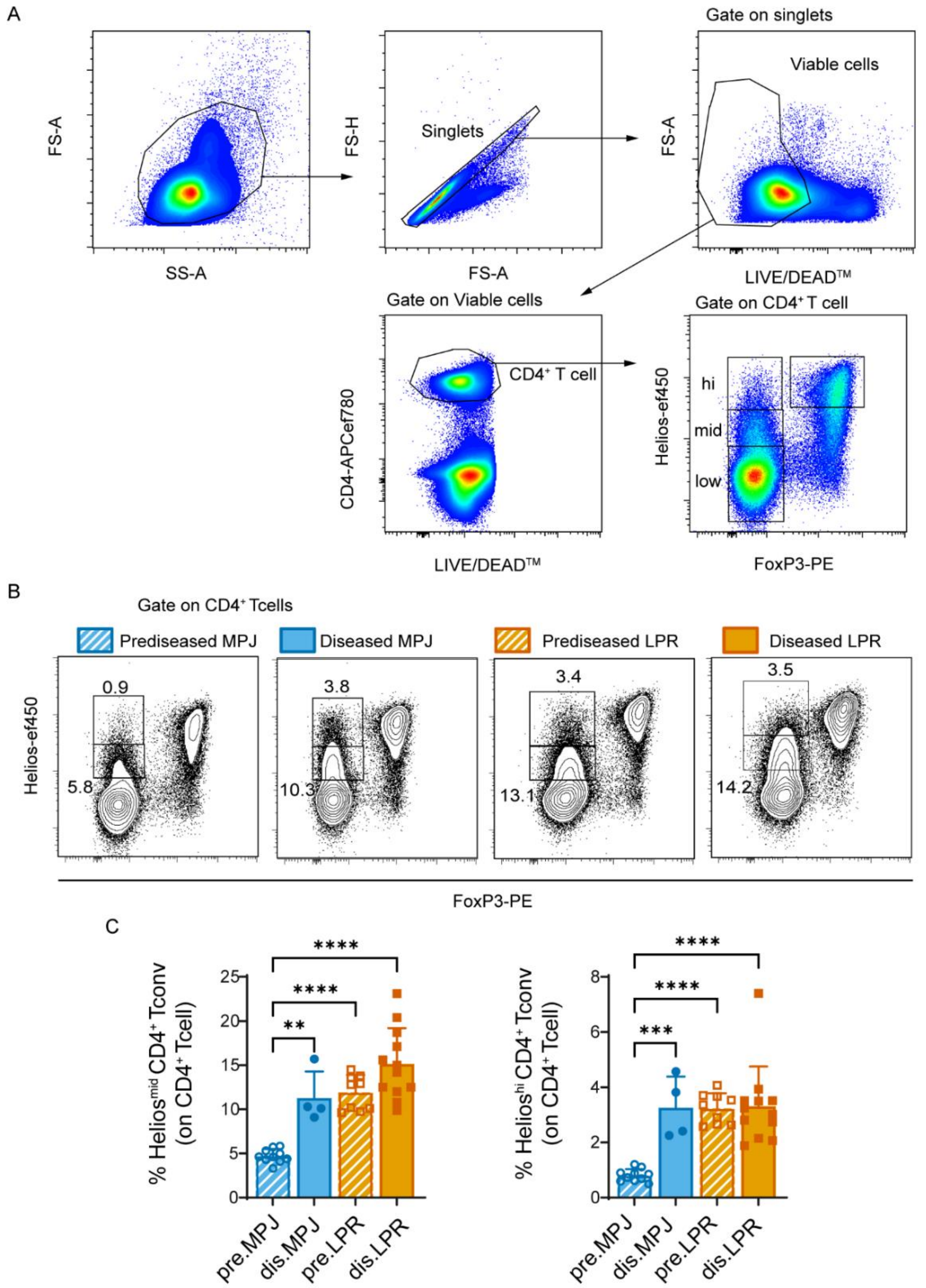


**Figure S2. Additional characterization of $CD4^+$ Tconvs from MPJ and LPR mice. (A)** Gating strategy used for splenic $CD4^+$ regulatory T (Treg) cells ($CD3^+CD4^+FoxP3^+Helios^{hi}$) and $CD4^+$ conventional T (Tconv) cells ($CD3^+CD4^+FoxP3^-$). Plots are from a prediseased MPJ mouse. **(B)** Representative flow cytometry plots gated on $CD4^+$ T cells, indicating the $Helios^{mid}$ and $Helios^{hi}$ $CD4^+$ Tconv populations. **(C)** Percentage of $Helios^{mid}$ (left) and $Helios^{hi}$ (right) $CD4^+$ Tconv among $CD4^+$ T cells. The data represent the mean ± SD: ** $P < 0.01$; *** $P < 0.001$; **** $P < 0.0001$ by one-way ANOVA with a Tukey multi-comparison post-test. Each point represents a single animal: prediseased MPJ (pre.MPJ), n = 10 (B and C), diseased MPJ (dis.MPJ), n = 4 (B and C); prediseased LPR (pre.LPR), n = 9 (B and C); diseased LPR (dis.LPR), n = 12 (B and C).

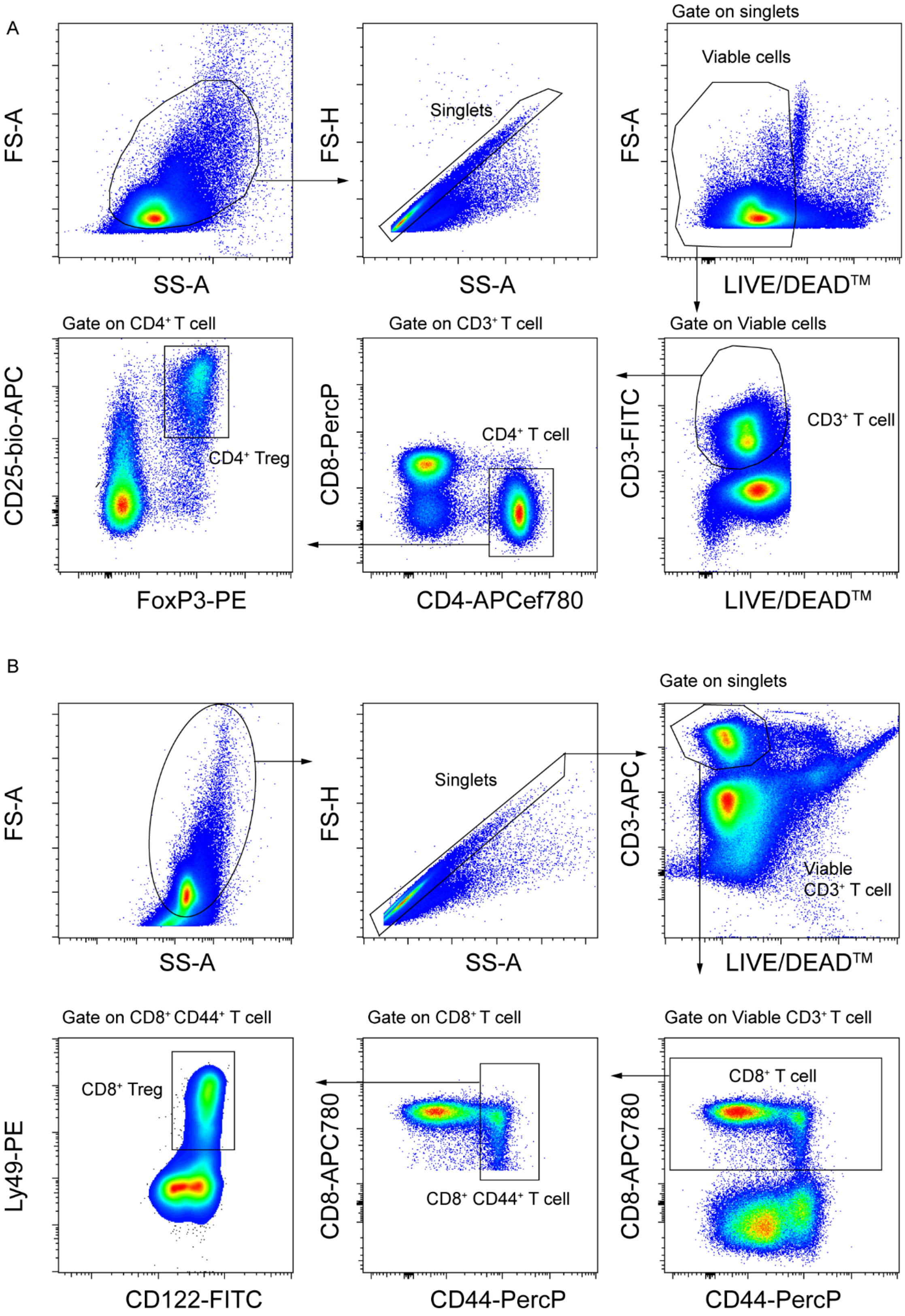


**Figure S3. Gating strategies for CD4$^+$ and CD8$^+$ Tregs from MPJ, LPR and C57BL/6 mice. (A)** Gating strategy used for splenic CD4$^+$ regulatory T (Treg) cells (CD3$^+$CD4$^+$CD8$^-$CD25$^+$FoxP3$^+$). **(B)** Gating strategy used for splenic CD8$^+$ regulatory T (Treg) cells (CD3$^+$CD8$^+$CD44$^+$CD122$^+$Ly49$^+$). The plots are from C57BL/6 mice.

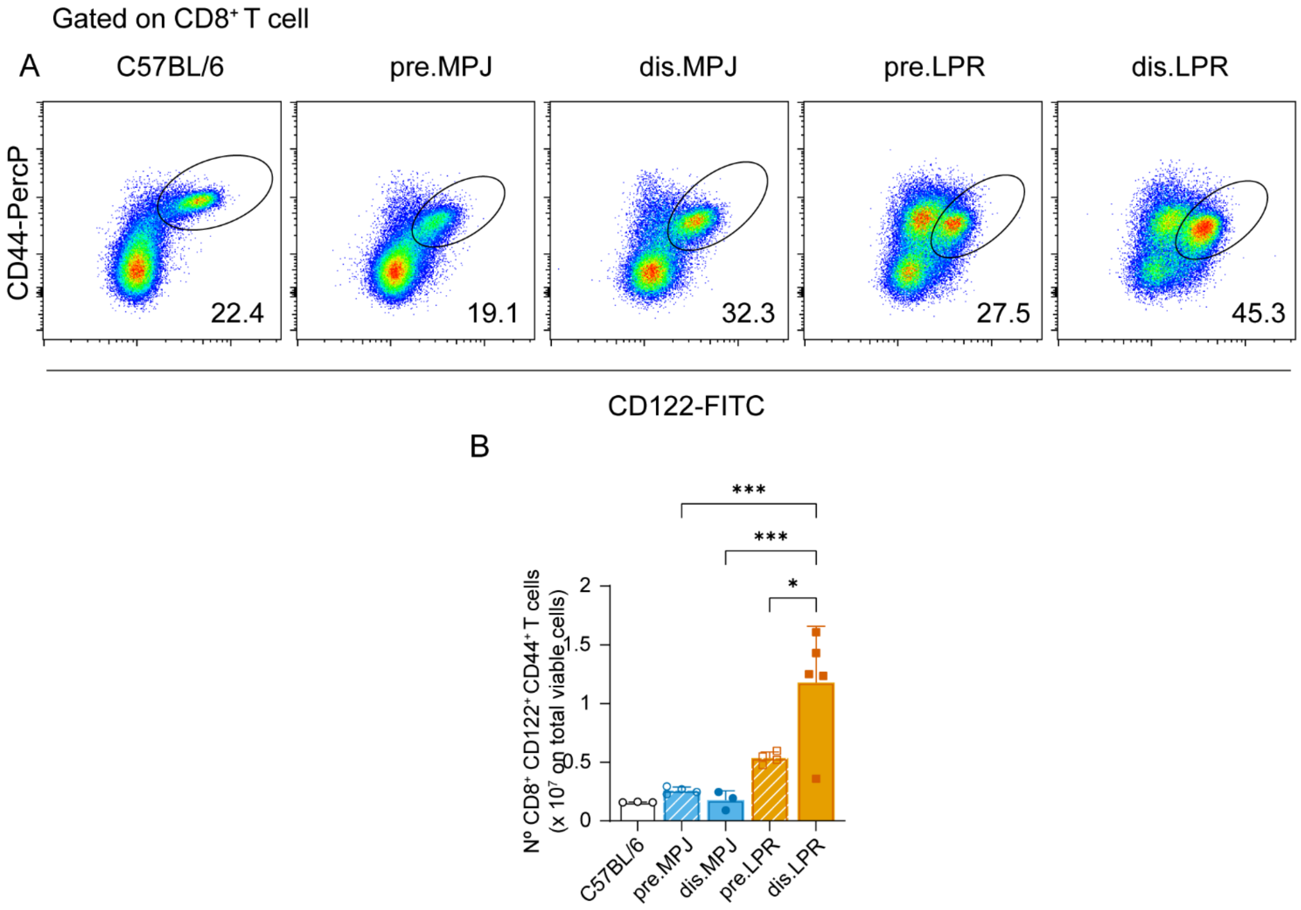


**Figure S4. Additional characterization of the CD8$^+$ T cells from MPJ, LPR and C57BL/6J mice. (A)** Representative flow cytometry plots gated on CD8$^+$ T cells, indicating the CD122$^+$ CD44$^+$ population. **(B)** Absolute number of CD8$^+$ CD122$^+$ CD44$^+$ T cells among total viable cells. The data represent the mean ± SD: * $P < 0.05$; *** $P < 0.001$; by one-way ANOVA with a Tukey multi-comparison post-test. Each point represents a single animal: C57BL/6, n = 3 (B), prediseased MPJ (pre.MPJ), n = 4 (B); diseased MPJ (dis.MPJ), n = 3 (B); prediseased LPR (pre.LPR), n = 4 (B); diseased LPR (dis.LPR), n = 5 (B).

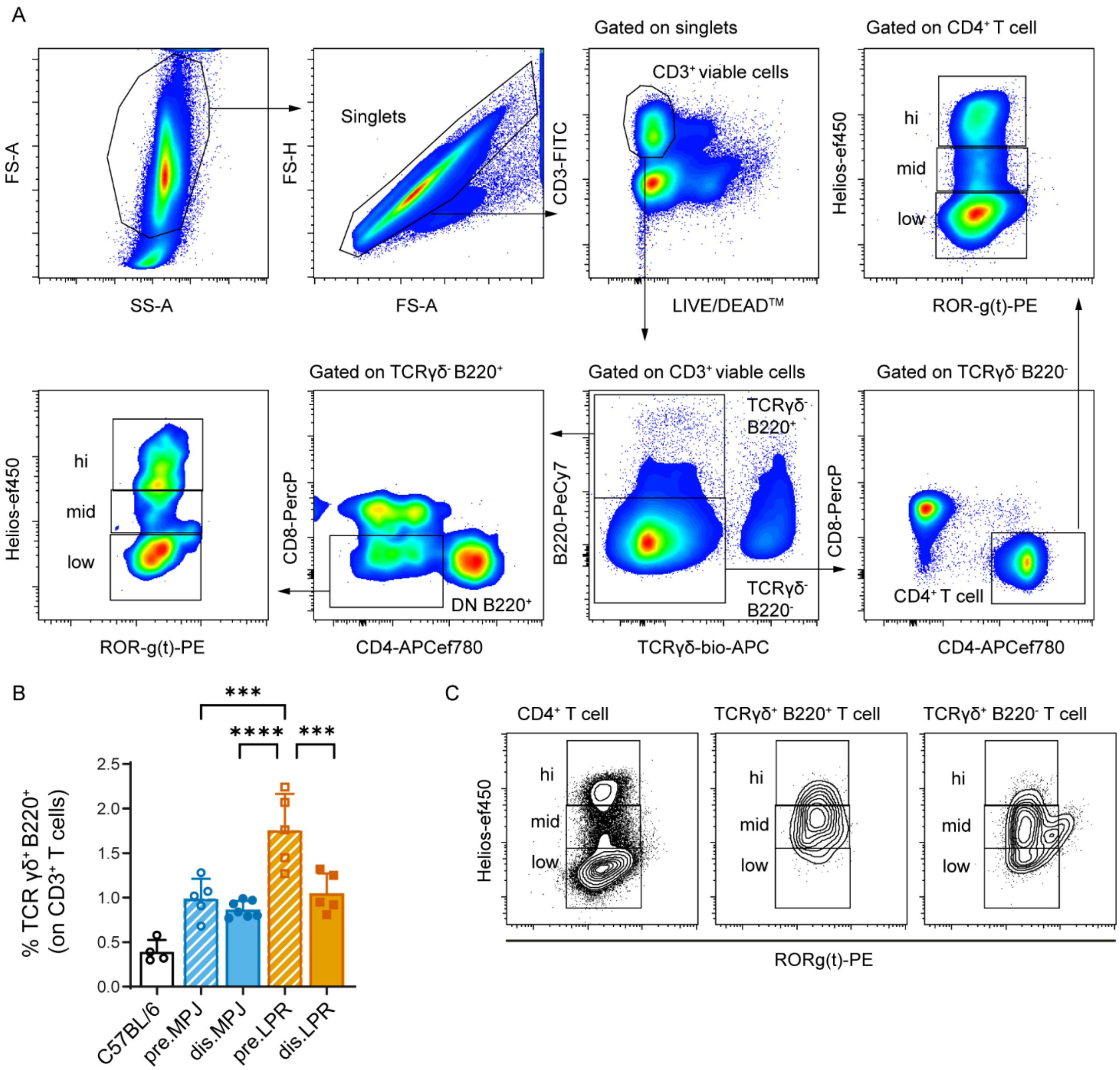


**Figure S5. Additional characterization of the DN and TCRγδ⁺ B220⁺ T cells from MPJ, LPR and C57BL/6J mice.** **(A)** Gating strategy used for splenic B220$^+$ DN T cells (CD3$^+$TCRγδ$^-$B220$^+$CD4$^-$CD8$^-$). Three different levels of Helios expression (Helios$^{hi/mid/low}$) were considered in CD4$^+$ T cells as a reference. **(B)** The proportion of TCRγδ$^+$ B220$^+$ T cells (CD3$^+$TCRγδ$^+$B220$^+$CD4$^-$CD8$^-$) among the total CD3$^+$ T cells. **(C)** Representative flow cytometry plots of CD4$^+$ (left) and TCRγδ$^+$ B220$^+$ T cells (middle) or TCRγδ$^+$ B220$^-$ (right) showing the Helios$^{hi/mid/low}$ gating. The data represent the mean ± SD: *** $P < 0.001$; **** $P < 0.0001$ for comparisons between MRL animals by a one-way ANOVA with a Tukey multi-comparison post-test. Each point represents a single animal: C57BL/6, n = 4 (B), prediseased MPJ (pre.MPJ), n = 5 (B); diseased MPJ (dis.MPJ), n = 6 (B); prediseased LPR (pre.LPR), n = 5 (B); diseased LPR (dis.LPR), n = 5 (B).

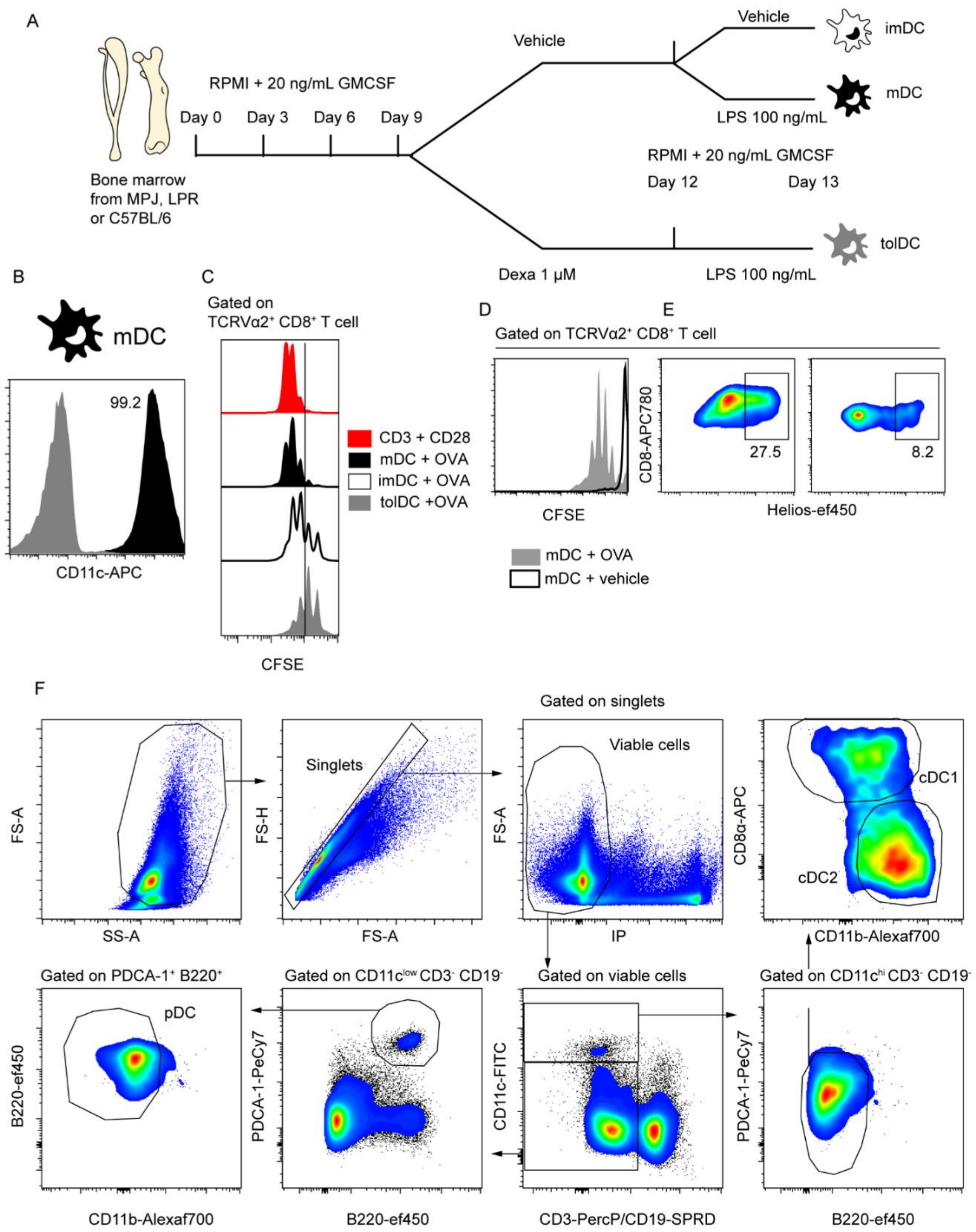


**Figure S6. Additional information regarding DCs. (A)** Scheme of the protocol to differentiate immature (imDC), mature (mDC) and tolerogenic (tolDC) dendritic cells (DCs) from hematopoietic precursors. **(B)** Representative CD11c histogram (black) of harvested mDCs on day 13 and their isotype control (grey). The proportion of $CD11c^{+}$ cells is indicated. **(C)** Representative CFSE histograms pregated on $TCRV\alpha2^{+}$ $CD8^{+}$ T cells from OT-I co-cultures after 3 days with mDCs, imDCs and tolDCs in the presence of OVA. Artificial CD3 and CD28 stimuli were used as a positive control of proliferation. **(D)** Histogram gated on $TCRV\alpha2^{+}$ $CD8^{+}$ T cells showing the CFSE signal of OT-I cells co-cultured with mDCs with or without OVA. **(E)** Helios expression on $TCRV\alpha2^{+}$ $CD8^{+}$ T cells from OTI splenocytes co-cultured with mDCs with or without OVA. **(F)** Gating strategy used for splenic plasmacytoid (pDCs: $CD11c^{low}CD11b^{-}B220^{+}PDCA\text{-}1^{+}CD3/CD19^{-}$), conventional type 1 (cDC1: $CD11c^{hi}CD11b^{-}CD8\alpha^{+}B220^{-}PDCA\text{-}1^{-}CD3/CD19^{-}$) and conventional type 2 (cDC2: $CD11c^{hi}$ $CD11b^{+}$ $CD8\alpha^{-}$ $B220^{-}$ $PDCA\text{-}1^{-}$ $CD3/CD19^{-}$). The plots are from a prediseased MPJ mouse.

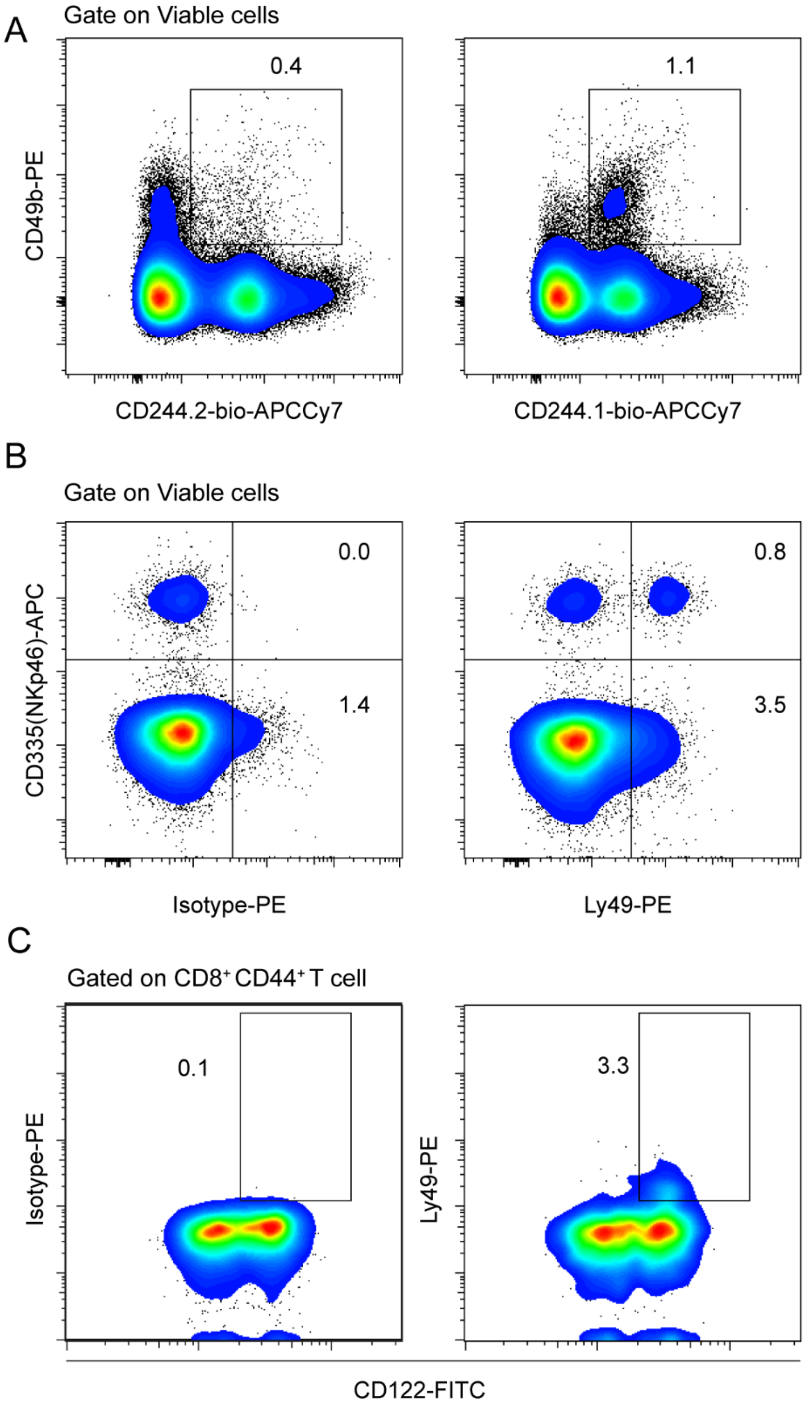


**Figure S7. Validation of antibodies for flow cytometry. (A)** Splenic cells were stained with anti-CD49b (DX5) and anti-CD244.2 (2B4) or anti-CD244.1 (REA524). CD49b$^+$ CD244$^+$ gate is showed. Plots are from a diseased LPR mouse. **(B)** Splenic cells were stained with anti-CD335(NKp46) and anti-Ly49C/I/F/H (14B11) or isotype control. Clone 14B11 shows a specific NK population in MRL genetic background. Plots are from a prediseased MPJ mouse. **(C)** Splenic cells were stained with anti-CD122 and anti-Ly49C/I/F/H (14B11) or isotype control. Clone 14B11 shows a specific population in MRL genetic background. Plots are from a diseased MPJ mouse.
